# Non-obvious Problems in Clark Electrode Application at Elevated Temperature and Ways of Their Elimination

**DOI:** 10.1155/2013/249752

**Published:** 2013-08-04

**Authors:** M. V. Miniaev, M. B. Belyakova, N. V. Kostiuk, D. V. Leshchenko, T. A. Fedotova

**Affiliations:** Research Center, Department of Chemistry and Biochemistry, and Department of Biology, Tver State Medical Academy, 4 Sovetskaya Street, Tver 170100, Russia

## Abstract

Well-known cause of frequent failures of closed oxygen sensors is the appearance of gas bubbles in the electrolyte. The problem is traditionally associated with insufficient sealing of the sensor that is not always true. Study of a typical temperature regime of measurement system based on Clark sensor showed that spontaneous release of the gas phase is a natural effect caused by periodic warming of the sensor to a temperature of the test liquid. The warming of the sensor together with the incubation medium causes oversaturation of electrolyte by dissolved gases and the allocation of gas bubbles. The lower rate of sensor heating in comparison with the medium reduces but does not eliminate the manifestation of this effect. It is experimentally established, that with each cycle of heating of measuring system up to 37°C followed by cooling the volume of gas phase in the electrolyte (KCl; 60 g/L; 400 **μ**L) increased by 0.6 **μ**L approximately. Thus, during just several cycles it can dramatically degrade the characteristics of the sensor. A method was developed in which the oxygen sensor is heated in contact with the liquid, (depleted of dissolved gases), allowing complete exclusion of the above-mentioned effect.

## 1. Introduction

Closed polarographic oxygen sensors (Clark electrodes and its varieties) are convenient and still the most widely used tools for measuring of oxygen consumption in various biochemical studies [1–4]. However, users of Clark electrode are often forced to face a number of problems, the causes of which are not obvious. Usually these are instability of readings, uniform signal drift, changes in sensor response time, formation of gas bubbles in electrolyte, and so forth. Unfortunately, these issues are not adequately addressed in the literature, as the results of failed measurements are not published, and the deviations are traditionally explained by damage of the membrane [5]. This greatly complicates the analysis of causes and, consequently, improvement of equipment and methods of measurement.

These problems are considerably aggravated when Clark electrode is applied for measurements in the heated solutions, even using an electronic temperature compensation [6]. For example, investigators of the current experiment found that after heating the incubation medium up to operating temperature (37°C) and saturating it by atmospheric oxygen, the results obtained during the first hour of work always significantly differed from the following ones. After several days of recurrent work at elevated temperature, the sensor showed a progressive decrease in its sensitivity. In some cases, the response time increased from the initial 30 seconds to 40 minutes, which was seen as a failure. Of course, these results have not been published.

When the failed sensor was disassembled, gas bubbles were always found in its electrolyte despite the fact that the integrity of the sensor was not broken, and the measurement of electrical resistance of the membrane demonstrated its intactness. The total volume of the gas bubbles could reach up to 1/4 of the electrolyte volume. This presumably caused the sensor failure [5, 7]. The bubbles appeared, even though the gas phase in the internal volume of the electrolyte was absent and carefully controlled at the assembly of sensor. It can be assumed that this is the particular reason why experiments with cells and mitochondria of warm-blooded organisms are usually conducted at room temperature [8, 9], regardless of the fact that ensuring stability at room temperature is technically more difficult than the optimum temperature for objects, 37°C.

In this aspect, the most essential constructive feature of Clark electrode is the presence of its own electrolyte volume that is separated by a gas-permeable polymer membrane from the analyzed liquid [10–12]. Oxygen is soluble in the incubation medium as well as in the electrolyte enclosed oxygen sensor [10]. For a number of reasons, the partial pressures of oxygen in these liquids may not coincide with each other. As a result, between liquids separated by membrane a diffusive gas exchange appears. At the same time, the current in the sensor (oximeter readings) depends on the rate of oxygen diffusion from the incubation medium to the cathode immersed in the electrolyte [10–12]. Therefore, any diffusion flow of oxygen through the membrane, not associated with the electrochemical processes at the cathode, will anyway affect oximeter readings.

Systematic observations of the behavior of the measuring system allowed to assume that the most probable cause of change of Po_2_ in the electrolyte may serve a heating or a cooling of the sensor [13–15]. For example, this occurs when the sensor is immersed in a solution heated up to 37°C. In this regard, it has been hypothesized that

1. during the warming of the measuring system, diffusional gas transfer occurs between its components;
2. restriction of diffusion by the sensor membrane leads to supersaturation of the electrolyte by dissolved gases and the formation of gas bubbles in it.

The following theoretical assumptions form the basis for acceptance of the hypothesis. At a constant temperature, the partial pressure *P* of the gas dissolved in the liquid in equilibrium with a gas phase is described by Henry's low [16]:

(1) $$\begin{array}{c} P=k_{H}\cdot c, \end{array}$$

where *P* is the partial pressure of gas in the solution, *c* is the concentration of gas in the solution in mole fraction, and *k*_*H*_ is Henry's constant.

When the temperature of a system changes, the Henry's constant will also change [17–19], according to the van't Hoff equation [20]:

(2) $$\begin{array}{c} k_{H}\left(T\right)=k_{H}\left(T_{298}\right)\cdot e^{-C\cdot (\left(1/T)-(1/T_{298}\right))}, \end{array}$$

where *k*_*H*_(*T*) is Henry's constant at temperature *T*, *k*_*H*_(*T*_298_) is Henry's constant that for the temperature 298 K, *C* is the constant characterized any certain gas, *T* is the absolute temperature of the solution, in *K*, *T*_298_ is the temperature 298 K, and *e* is constant that is the base of the natural logarithm.

**Figure 1:**
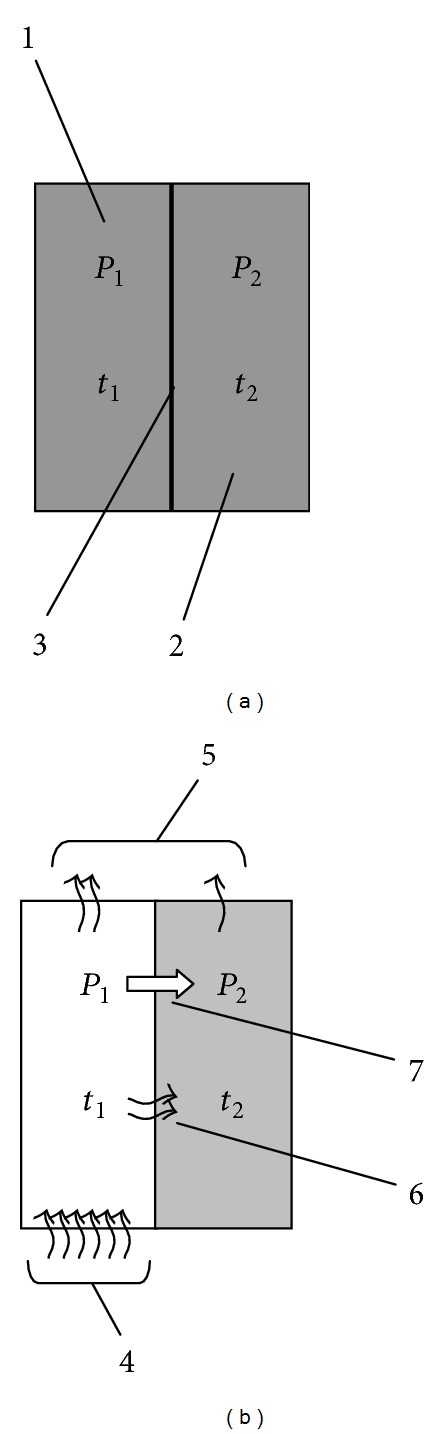
Change of the partial pressure of the gas in solutions separated by a gas-permeable membrane during the forced heating of one of them: (a) initial equilibrium state (*t*_1_ = *t*_2_ = Const, *P*_1_ = *P*_2_ = Const), (b) heating (*t*_1_ > *t*_2_; *P*_1_ > *P*_2_). The wavy arrows are heat transfer; white arrows are the diffusion of the dissolved gas. (1) is the first liquid, (2) is second liquid, (3) is gas-permeable membrane, (4) is the heating of the first fluid from an external source of heat, (5) is heat loss, (6) is heating of the second liquid through the membrane, and (7) is diffusion of dissolved gases. The temperature of the first liquid which (1) is heated from the outside (4) increases faster than temperature of the second (2), and therefore the partial pressure of gases dissolved in the first liquid is also increasing rapidly. The resulting difference in partial pressures causes diffusion of dissolved gases (7) from the first liquid into the second through gas-permeable membrane separating them (3).

It follows that a rapid increase in temperature of the liquid at complicated gas exchange with the environment will elevate partial pressures of dissolved gases. Thus, if one of the two liquids separated by a membrane (Figure 1) is heated by the heat supply from the outside, and the other is through contact with the first, the rate of warming-up of the first is greater. As a consequence, the partial pressure of dissolved gas in the first liquid will also grow faster, which results in a partial pressure difference Δ*P* on the membrane separating the liquid:

(3) $$\begin{array}{c} \Delta P=P_{1}-P_{2}, \end{array}$$

where *P*_1_ is the partial pressure of the gas in the warmed-up solution and *P*_2_ is the partial pressure of the gas in the cold solution.

If the membrane is gas permeable, it will cause the diffusion of dissolved gases through the membrane in the direction of warming-up (Figure 1(b)), according to Fick's law of diffusion for inhomogeneous media [21]:

(4) $$\begin{array}{c} \frac{dm}{dt}=\frac{DS\;\Delta P}{\delta }, \end{array}$$

where *S* is cross-sectional area of the solution, through which the diffusion occurs, Δ*P* is the partial pressure difference, *D* is diffusion coefficient, and *δ* is thickness of the diffusion layer.

The direction of the dissolved gas diffusion in this case will not be determined by the value of the temperature difference of contacting liquids but the difference in the rates of their warming-up. The gas will diffuse out from the rapidly heated liquid into a liquid with lower rate of warming. For this reason, termination of the first liquid warming will cause the change of the direction of the dissolved gas diffusion to reverse despite the fact that for some time its temperature will be higher than the temperature of the second liquid, which will continue to warm due to contact with the first one. If the permeability of the membrane that separates the liquids is insufficient to ensure full diffusion flow of dissolved gas, the formation of gas bubbles must be observed in the second liquid, because the capacity of the bubbles for atmospheric gases is much higher than of electrolyte solution of the same volume [17].

## 2. Methods

### 2.1. Measuring System

**Figure 2:**
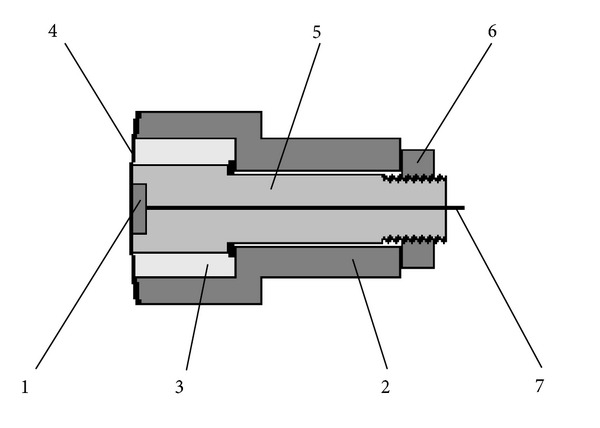
Closed oxygen sensor: (1) is cathode (Ag; 5 mm^2^), (2) is the body-anode (Zn; 300 mm^2^), (3) is electrolyte (KCl; 60 g/L, 400 *μ*L), (4) is membrane (PE; 50 *μ*m, 100 mm^2^), (5) is the body of the cathode (polymethyl methacrylate, PMMA), (6) is nut, and (7) is output to oximeter. The main feature of this oxygen sensor is accelerated gas exchange between the test liquid and electrolyte (3) through a polyethylene membrane (4). A small amount of electrolyte, the location of the total volume as close as possible to the membrane, and a large area of the membrane contribute the acceleration.

**Figure 3:**
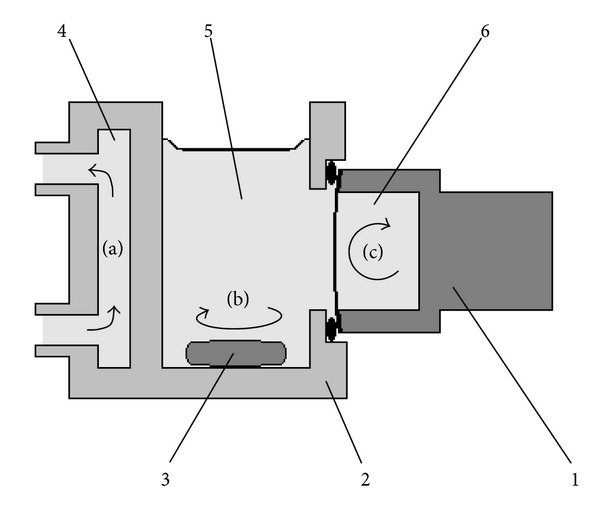
Thermostated measuring cell: (1) is oxygen sensor (Figure 2, a cathode is not shown), (2) is measuring cell (PMMA), (3) is magnetic stirrer, (4) is heat carrier from thermostat, (5) is conditional incubation medium (KCl; 60 g/L, 3.5 mL), and (6) is electrolyte of the sensor (KCl; 60 g/L, 400 *μ*L). (a) is circulation of heat carrier, (b) is mixing of the medium by a magnetic stirrer, and (c) is convective mixing of the electrolyte due to a temperature difference between the membrane and the sensor's body. The temperature gradient between the heated incubation medium (5) and colder electrolyte (6) of oxygen sensor (1) causes convective mixing of the electrolyte (c). This significantly accelerates the gas exchange between the electrolyte and the medium [23].

We used measuring system, consisting of an oxygen sensor (Figure 2), which was a Clark electrode [11] in the modification of Mancy [22] and a thermostated measuring cell (Figure 3) with side mount of the sensor.

Sensor's readings were recorded by an oximeter N5221 (Poland). Oximeter was used only to control the residual partial pressure of oxygen in the washing liquid, so sensor's readings during operating were not recorded.

### 2.2. Conditional Medium for Incubation and Washing Liquid

Any object consumed oxygen was not used in the work, so the liquid that filled the measuring cell was considered as a conditional medium for incubation. The base for the preparation of both the medium and the washing liquid was a solution identical to the electrolyte of closed oxygen sensor (KCl; 60 g/L), that allowed to avoid the osmotic by-effects and simplify the interpretation of results. To prepare the medium, initial solution was saturated with atmospheric oxygen by continuously blowing air in the vessel, thermostated at 37°C. The partial pressure of oxygen during the work in the medium was not changed (100% on the scale of the oximeter). The volume of the medium which was poured into the measuring cell in all cases was 3.5 mL.

To prepare the washing liquid, the same solution was placed in a thermostated flask at 37°C and kept there during the experiment at low pressure developed by continuously operating peristaltic pump. The content of dissolved gases in the liquid was controlled on a residual oxygen partial pressure, which ranged from 5 to 15% on the scale of the oximeter. Added volume of washing liquid in all cases was 3.5 mL.

### 2.3. Temperature Mode of Measuring System

**Figure 4:**
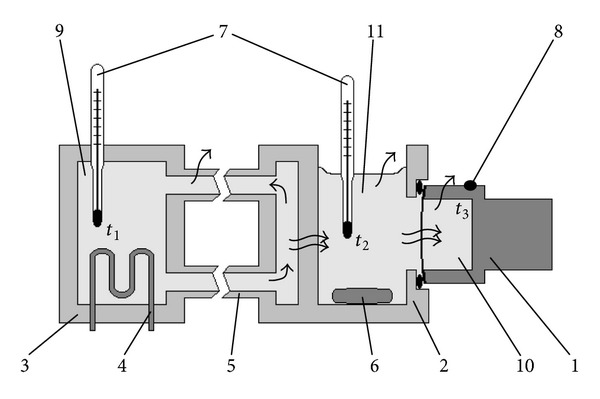
Scheme of warming-up (wavy arrows are heat transfer) of the measuring cell up to the operating temperature: (1) is oxygen sensor (cathode is not shown), (2) is measuring cell, (3) is thermostat, (4) is heating element, (5) is connecting tubes, (6) is magnetic stirrer, (7) is thermometers, (8) is mounting place of thermocouples, (9) is heat carrier, (10) is electrolyte of oxygen sensor, and (11) is incubation medium. *t*_1_, *t*_2_, and *t*_3_ are points of temperature control. Warming-up of the system is carried out in the direction of the thermostat-medium-electrolyte. In the state of thermal equilibrium, temperature of the incubation medium (11) is appreciably below the temperature of the heat carrier (9) into the thermostat (3) due to heat losses in the connecting tubes (5). The temperature of the electrolyte (10) is even lower due to the cooling of the oxygen sensor body (1) by ambient air.

The system was thermostated at 37°C (on the incubation medium) using the flow thermostat (±0.1°C) during operation, and in the intervals between cycles warming-up/cooling was at room temperature (20–25°C). Because of heat loss, the operating temperature of the system was set up as a state of equilibrium between the temperature of its components and the ambient temperature. Herewith warming-up of the sensor was carried out through the incubation medium, the electrolyte, and the membrane (Figure 4).

Two laboratory thermometers (±0.5°C) selected according to equality of readings were used to control the temperature of the heat carrier of thermostat (*t*_1_) and incubation medium (*t*_2_). Temperature of the zinc body in the oxygen sensor (*t*_3_) was controlled by calibrated thermocouple in conjunction with a digital multimeter MS8201H (Korea). Thermometers were directly immersed in the appropriate liquids and a thermocouple was attached to the metal body of the sensor by a hot-melt adhesive (Figure 4).

Thermostating of the oxygen sensor was abandoned in order to accelerate gas exchange between the electrolyte and the incubation medium. Acceleration was achieved by convection currents in the electrolyte (Figure 3(c)) due to the difference in temperature between the sensor body and its membrane. As a consequence, before each measurement a relatively rapid stabilization of the partial pressure of oxygen in the electrolyte sensor is achieved. As a result, the coefficient of value variation, obtained by dynamic measurements of the oxygen absorption by sodium sulfite [23], was reduced to 1.5% (coefficient of variation in concentration of sodium sulfite is 1.3%). In addition, deceleration of warming the oxygen sensor, during preparation of the system for operation, to some extent had to impede the oversaturation of electrolyte by dissolved atmospheric gases. Finally, in such conditions work the most common measuring systems equipped with submersible sensors, not mounted in the thermostated body of measuring cell [5, 11, 22].

## 3. Results and Discussion

### 3.1. Dynamics of Warming the Measuring System Components

According to the hypothesis, the unequal rates of the heating of the measuring system's components are the most probable reason of the diffusive flow of oxygen through the membrane of the sensor. Therefore we studied the dynamics of warming-up of the components and determined their equilibrium temperature.

For this purpose 6 cycles of heating the measuring system from room (20–25°C) up to operating (37°C) temperature were carried out. During the cycle, the temperature of components was recorded every 10 minutes from the time the thermostat started to work (0 min). Between cycles, the system was kept overnight at room temperature. The results are shown in Table 1.

Rate of warming-up of individual components of the measurement system varied. Equilibrium temperature of the thermostat was achieved after 30 minutes of warming and was 39.2°C. Warming-up the incubation medium, in the measuring cell, to the equilibrium temperature of 36.9°C took 90 minutes. The body of the oxygen sensor heated most slowly (130 min). Its temperature was 35.3°C at equilibrium state. The temperature difference (Δ*t*) between the incubation medium and the body of the sensor, sustainable for convection of the electrolyte, was equal to 1.6°C in equilibrium.

Thus, the preconditions for gas exchange between the incubation medium and the electrolyte of the oxygen sensor retained for at least 130 minutes from the start of warming-up, and for 40 min after the moment of temperature stabilization in the incubation medium.

### 3.2. The Dynamics of the Temperature Drop “Medium-Electrolyte”

**Figure 5:**
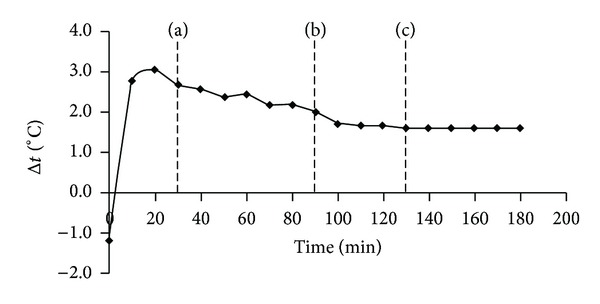
Dynamics of temperature difference between the incubation medium and the oxygen sensor electrolyte (a) at temperature equilibrium of the thermostat; (b) at temperature equilibrium of the incubation medium; and (c) at temperature equilibrium of the sensor electrolyte. Electrolyte of sensor clearly begins to warm up before stabilization of the thermostat's temperature (a). Because rate of thermostat's heating near the temperature of the thermostating is sharply slowed down, speed of the electrolyte's warming-up exceeds the rate of warming up of the medium from 20 to 130 min (c) from the moment of switching the thermostat. Thus, conditions for the diffusion of gases from the sensor to the incubation medium remain over 110 min. Stabilization of the medium temperature (b) does not render appreciable influence on this process.

To determine the probable directions of gas flows, it was necessary to characterize the dynamics of the temperature difference between the incubation medium and the electrolyte of the closed oxygen sensor, in the process of warming the measuring system up to thermal equilibrium. As presented in Figure 5, the difference is sharply increased during the forced warming-up of the thermostat and reached a maximum after 20 minutes. As the temperature of the incubation medium approached incubation temperature (37°C), and rate of medium warming-up decreased, the temperature difference Δ*t* began to be determined by the warming-up process of the oxygen sensor electrolyte. As a result, since 30 minutes from the time the thermostat was switched on, the difference also began to decline. The decrease gradually slowed down and the difference reached its equilibrium value of 1.6°C in 130 minutes.

Thus, for 130 min from the beginning of heating of the measuring system, the temperature difference between the incubation medium and the sensor electrolyte goes through three successive stages: increasing (0–20 min), decreasing (20–130 min), and stabilization (over 130 min). The diffusion of gases from the incubation medium into the sensor electrolyte should be observed, at the first stage (that should further contribute to supersaturation of the electrolyte by dissolved gases), and from the electrolyte into the medium at least up to the state of thermal equilibrium, at the second stage.

### 3.3. Experimental Verification of the Hypothesis

**Figure 6:**
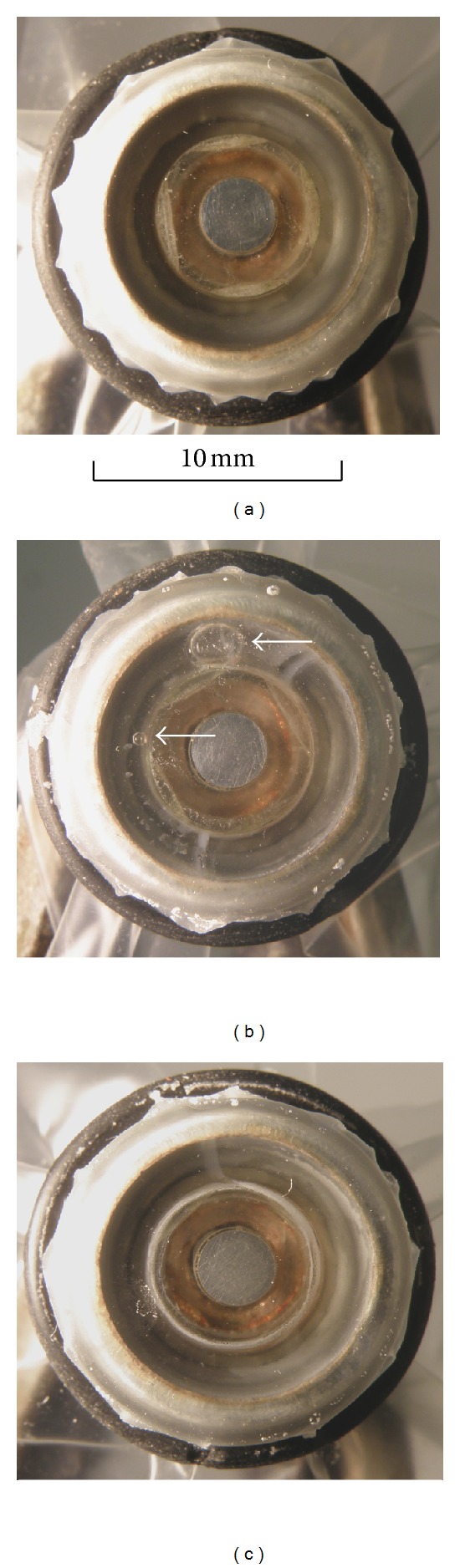
States of the closed oxygen sensor before and after four cycles of warming-up/cooling (gas bubbles are indicated by arrows): (a) initial state, (b) after the use of medium saturated with atmospheric air, (c) after the use of medium depleted of dissolved gases. A view of the sensor with the cathode side is shown. For 4-cycle heating/cooling in all cases (b, c) there is negligible corrosion of the zinc anode and the appearance of salt deposits on the inner side of the membrane. If the heating takes place in medium saturated with atmospheric oxygen (b), the changes are more pronounced and in addition the formation of gas bubbles is observed. When heating is carried out in the medium depleted of dissolved gases (c), after the experiment, condition of the sensor does not differ from the initial (a).

To confirm the hypothesis and to estimate approximately the amount of atmospheric gases diffused through the membrane at warming up of the sensor, 4 cycles heating/cooling of the measuring system were held that simulated a work with the sensor at a temperature of 37°C. Assembled sensor was carefully examined for the absence of gas bubbles in the electrolyte and its original state was fixed by photography before the series of cycles (Figure 6(a)). 

Cycles were carried out one per day. In each cycle, the system was heated for 140 minutes up to 37°C, after that termostating was continued at the operating temperature for 1 hour. After work the thermostat was turned off, the measuring cell was filled with fresh incubation medium and was kept at room temperature. The next day the cycle was repeated.

When four cycles were completed, a two air bubbles were revealed in the sensor electrolyte (Figure 6(b)). Approximate volume of the bubbles was calculated from their linear dimensions in the photo considering the zoom scale. Due to the fact that the rigidity of the membrane is negligible, the compressibility of the gas was not taken into account. The total volume of bubbles at 20°C, calculated on the assumption that each of them had a regular spherical shape, was about 2.5 *μ*L. Hence it follows that in every cycle heating/cooling the volume of air bubbles in the electrolyte of the closed oxygen sensor has increased by approximately 0.6 *μ*L.

If we consider that in the sensor electrolyte (760 mmHg, 400 *μ*L of KCl solution, 60 g/L) it may be dissolved approximately 4.8 *μ*L of air at 20°C, or 3.2 *μ*L at 40°C, than 1.6 *μ*L of the air gases was released from the electrolyte due to each act of warming-up. Approximately 60% of this diffused to the incubation medium and then into the atmosphere, and the remaining 40% (0.6 *μ*L) was delayed by membrane and replenish the gas phase in the compartment of the sensor (Figure 6(b)).

Thus, periodic work of the closed oxygen sensor at higher temperature is actually accompanied with gas exchange between the components of the measurement system. Although flow of gas is relatively small, it may introduce a noticeable distortion in the work of measuring system for at least 40 minutes from completed warm-up of the measuring cell to operating temperature. Repetition of the cycles heating/cooling inevitably leads to a rapid sensor failure due to accumulation of gas in the electrolyte of the oxygen sensor.

### 3.4. The Proposed Mechanism of Gas Formation in Electrolyte

**Figure 7:**
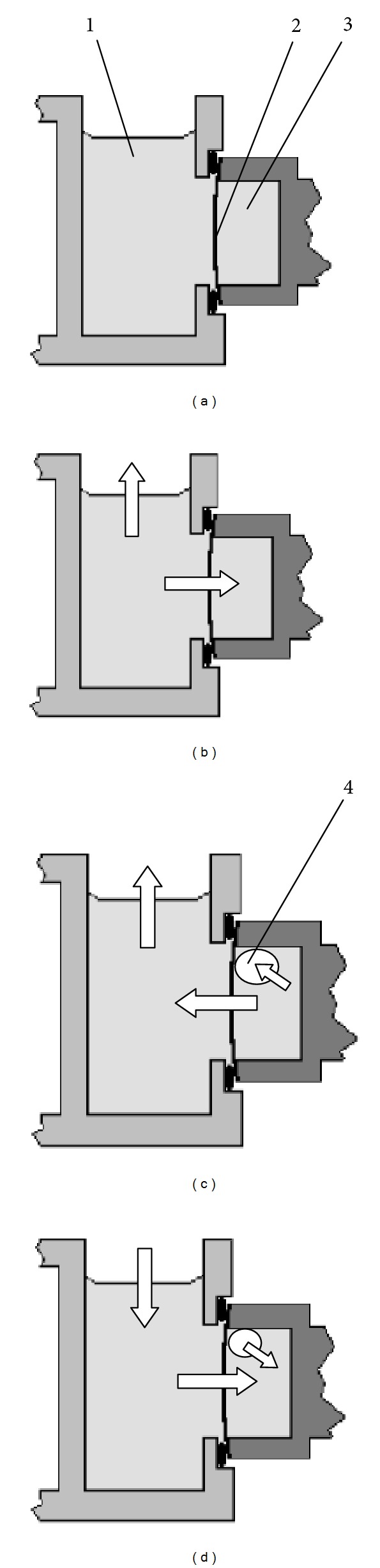
The proposed mechanism of gas bubbles formation in the electrolyte at periodic heating/cooling of the oxygen sensor (diffusion fluxes of gases are shown by arrows): (a) is initial state of the system at room temperature, (b) is rapid heating of the incubation medium, (c) is heating of the oxygen sensor after thermostabilization of the incubation medium, and (d) is cooling of the systems up to room temperature after work. (1) is the incubation medium, (2) is membrane, (3) is sensor electrolyte, and (4) is gas bubble. The main reason for the formation of bubbles (4) is supersaturation of the electrolyte (3) of the oxygen sensor during the heating (b, c) with insufficient rate of gas exchange with a medium (1) limited by a permeability of membrane (2). At the same time, even the low permeability of the membrane allows gases to return into the electrolyte from the medium under cooling (d). For this reason, bubbles are not dissolved completely under cooling.

The obtained results allow suggesting a possible mechanism of gas bubbles formation in the electrolyte of the oxygen sensor. It was assumed that in the incubation medium (or another liquid that fills the cell during storage) and in the electrolyte of the oxygen sensor, kept for more than 20 hours at room temperature, partial pressures of dissolved gases are equal to their partial pressures in the atmosphere. Therefore, gas exchange between the liquids themselves as well as with the atmosphere does not occur initially (Figure 7(a)).

At startup of thermostat, the incubation medium warms up quickly, so partial pressures of dissolved gases increase. As a result, at this stage they should diffuse into a region with lower partial pressure: into the atmosphere, where partial pressure of gases is constant, and into the electrolyte of the oxygen sensor which is warmed up much slower than the medium (Figure 7(b)).

As the temperature of the incubation medium approaches to the thermostating temperature, the rate of its warming-up decreases rapidly, and partial pressures of dissolved gases becomes almost even with their partial pressures in the atmosphere. At this time, the electrolyte continues to warm, that is accompanied by a further increase in the partial pressure of dissolved gases in it. As a result, the gases at this stage should diffuse from the electrolyte into the incubation medium, and further into the atmosphere. Since the membrane has a certain resistance to the diffusion [12, 24], part of the gases can be released directly into the electrolyte in the form of gas bubbles (Figure 7(c)). Diffusion of gases from the electrolyte into the medium and the formation of bubbles should be stopped not earlier than the temperature of the electrolyte of the oxygen sensor reaches an equilibrium value that is approximately two hours after startup of the thermostat.

The subsequent slow cooling of the system up to room temperature at the end of the work should not lead to the complete dissolution of the bubbles because a part of the gases will go back into the electrolyte through the medium and the membrane from the atmosphere (Figure 7(d)). Hence, with each new cycle heating/cooling (with each working day) the volume of the gas phase in the compartment of sensor should increase.

### 3.5. Experimental Verification of Proposed Mechanism of Gas Formation

Thus, the immediate cause of formation of gas bubbles is presumably insufficient rate of gas exchange through the membrane between the incubation medium and the electrolyte of the oxygen sensor. Therefore, to confirm this hypothesis it was decided to accelerate the diffusion by increasing of the partial pressure difference of dissolved gases between the electrolyte and the medium. To do this, at warming-up the measuring cell was filled by periodically refreshed washing liquid, depleted in oxygen and nitrogen.

4 cycles of warming-up/cooling were performed, similar to what was described above but with the following changes:

1. prior to warming-up, the measuring cell was filled twice with washing liquid which was kept in a cell until the oximeter readings reached 40%;
2. before turning on the thermostat, the cell was filled with fresh washing liquid;
3. washing liquid was replaced in the cell with the following intervals during warming-up: at temperature below 30°C—liquid was kept in the measuring cell until the oximeter readings increase up to 40%; at temperatures of 30 to 35°C—while the ones reached 60%; and in the range of 35 to 37°C—up to the 80%, correspondingly;
4. when the equilibrium temperature was established, the washing liquid was replaced by the incubation medium saturated with atmospheric oxygen, and then the system was left to stand for 30 minutes before using the sensor;
5. after work (1 hour of periodic replacement of the incubation medium) the cell was refilled with fresh washing liquid and left at room temperature. The next day the cycle was repeated.

The results are presented in Figure 6(c). As shown, the sensor initially not contained gas phase was free of gas bubbles upon completion of four warming-up cycles. Thus, the proposed mechanism quite accurately reflects the processes occurred in a closed oxygen sensor at periodic heating-cooling.

## 4. Conclusions

1. Heating of the closed oxygen sensor up to operating temperature is accompanied by intensive exchange of gases through the membrane between the sensor electrolyte and the incubation medium. The direction of gas diffusion can be changed to the opposite depending on the stage of warming-up.
2. Gas exchange arises from the beginning of the elevation of the incubation medium temperature and ends up not earlier than one hour after its stabilization. Therefore, stabilization of the medium temperature is not an indicator of readiness to work of measurement system.
3. The resistance of the membrane for atmospheric gases is too large to transmit the entire gas flow from the warmed electrolyte to the incubation medium. Therefore, dissolved gases form bubbles in supersaturated electrolyte.
4. Filling the measuring cell by sodium sulfite solution or other oxygen scavengers at the time of storage does not solve the problem of gas formation because it does not prevent saturation of the electrolyte with atmospheric nitrogen. Thermostating the oxygen sensor coupled with the measuring cell seems ineffective too. This can only accelerate supersaturation of electrolyte by dissolved gases at warming-up and enhance the formation of gas bubbles.
5. Gas formation and thus the rapid failure of the oxygen sensor can be avoided by using the method of its warming up to operate temperature in permanent contact with the liquid where the partial pressure of dissolved gases (including nitrogen) is significantly lower than in the sensor electrolyte.
6. The revealed regularities are common to any measuring systems of this type.

## Figures and Tables

**Table 1:** Dynamics of warming-up of the measuring system's components *(n* = 6).

Time (min)	Temperature (°C)					
	*t*_1_	± SE	*t*_2_	± SE	*t*_3_	± SE
0	21.9	1.2	24.4	0.4	25.6	0.4
10	34.6	1.6	31.0	0.3	28.2	0.4
20	39.0	0.1	34.7	0.2	31.6	0.3
30*	39.2	0.0	35.8	0.2	33.1	0.3
40	39.2	0.0	36.1	0.2	33.6	0.2
50	39.2	0.0	36.5	0.2	34.1	0.4
60	39.2	0.0	36.8	0.2	34.3	0.4
70	39.2	0.0	36.8	0.2	34.6	0.2
80	39.2	0.0	36.8	0.2	34.6	0.2
90**	39.2	0.0	36.9	0.2	34.8	0.2
100	39.2	0.0	36.9	0.2	35.1	0.3
110	39.2	0.0	36.9	0.2	35.2	0.4
120	39.2	0.0	36.9	0.2	35.2	0.4
130***	39.2	0.0	36.9	0.2	35.3	0.4
140	39.2	0.0	36.9	0.2	35.3	0.4
150	39.2	0.0	36.9	0.2	35.3	0.4
160	39.2	0.0	36.9	0.2	35.3	0.4
170	39.2	0.0	36.9	0.2	35.3	0.4
180	39.2	0.0	36.9	0.2	35.3	0.4

*Time of temperature stabilization of the thermostat heat carrier.

**Time of temperature stabilization of the incubation medium.

***Time of temperature stabilization of the oxygen sensor body.
